# The Role of Heritable Tumors in Evolution of Development: a New Theory of Carcino-evo-devo

**DOI:** 10.32607/20758251-2019-11-4-65-72

**Published:** 2019

**Authors:** A. P. Kozlov

**Affiliations:** Vavilov Institute of General Genetics RAS, Moscow, 119333 Russia Biomedical Center, Research Institute of Ultrapure Biologicals and Peter the Great St. Petersburg Polytechnic University, St. Petersburg, 195251 Russia

**Keywords:** heritable tumors, embryonic development, evo-devo, carcino-evo-devo

## Abstract

The hypothesis of evolution by tumor neofunctionalization (the "main hypothesis") describes the possible role of hereditary tumors in evolution. The present article examines the relationship of the main hypothesis to other biological theories. As shown in this paper, the main hypothesis does not contradict to the existing biological theories, but fills the lacunas between them and explains some unexplained (or not completely understood) questions. Common features of embryonic development and tumorigenesis are described by several recognized theories. Similarities between normal development and tumorigenesis suggest that tumors could participate in the evolution of ontogenesis and in the origin of new cell types, tissues and organs. A wide spectrum of non-trivial explanations and non-trivial predictions in different fields of biology, suggested by the main hypothesis, is an indication of its fundamental nature and the potential to become a new biological theory, a theory of the role of tumors in evolution of development, or *carcino-evo-devo*.

## INTRODUCTION

Multicellular organisms needed a continuous source of additional cell masses with high biosynthetic and morphogenetic potential as a material for progressive evolution, especially in the line Deuterostomia – Chordata – Vertebrata. The problem of the origin of such cell masses has not been resolved. It is clear that stem cells should participate in this process, but adult and embryonic stem cells are regulated by functional feedback loops and cannot provide considerable amounts of excessive cells. Physiological proliferative processes existing in normal organisms could not provide sizeable extra cell masses because such proliferative processes are functional and are regulated with feedback loops.

On the other hand, tumors and tumor stem cells are not (or less) regulated and potentially could provide the evolving multicellular organisms with unlimited amounts of extra cells with high biosynthetic and morphogenetic potential.

The hypothesis of evolution by tumor neofunctionalization (below I will call it "the main hypothesis") suggests that the possible role of hereditary tumors in evolution might consist in providing extra cell masses for the expression of evolutionarily novel genes and gene combinations, and for the origin of new cell types, tissues and organs [1]. The main hypothesis formulated several non-trivial predictions; some of them have already received experimental confirmation [1-3]. In the present article, I will examine the relationship of the main hypothesis to other biological theories.

## NON-TRIVIAL EXPLANATIONS OF THE MAIN HYPOTHESIS AND ITS RELATIONSHIP TO OTHER BIOLOGICAL THEORIES

**Fig. 1:**
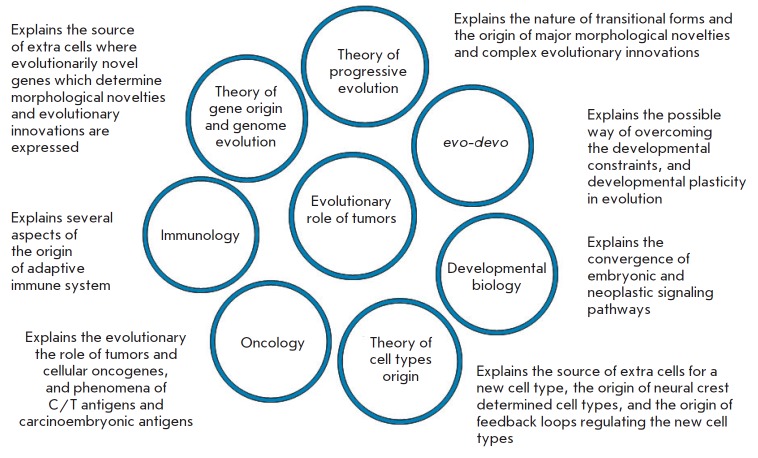
Non-trivial explanations of the main hypothesis and its relationships to other biological theories

The main hypothesis does not contradict the existing biological theories but fills the lacunas between them and explains some unexplained (or not completely understood) questions *(Fig. 1)*. Explanation of the phenomena unexplained on not completely explained by the pre-existing theories, together with non-trivial predictions, is the fundamental demand to the new scientific theory.

In theory of progressive evolution, the main hypothesis explains the nature of transitional forms, and the origins of complexity. It explains the possible mechanism of the origin of major morphological novelties such as evolutionarily new organs and complex evolutionary innovations such as the adaptive immune system.

In *evo-devo*, the main hypothesis explicates the possible way to overcome developmental constraints, and the mechanism of developmental plasticity in progressive evolution. It also suggests the neoplastic mode of evolution of ontogenesis.

In developmental biology, this hypothesis offers an explanation for the convergence of embryonic and neoplastic signaling pathways.

In the theory of cell types origin, it explains the source of extra cells for a new cell type, the origin of neural crest determined cell types, and the origin of feedback loops regulating the new cell types. The role of oncogenes, tumor suppressor genes, and novel genes and gene combinations in the origin of new cell types is also explained.

In the theory of gene origin and genome evolution, it offers an explanation for the source of extra cells where the evolutionarily novel genes determining the morphological novelties and evolutionary innovations are expressed.

In oncology, it construes the evolutionary role of tumors and cellular oncogenes, phenomena of cancer/testis antigens and carcinoembryonic antigens, etc.

In immunology, the main hypothesis explains several aspects of the origin of the adaptive immune system.

Non-trivial explanations offered by the main hypothesis were well accepted by representatives of corresponding branches of biological science during a number of my presentations to different audiences.

The explanations being most important for the present paper are those of the problem of transitional forms in progressive evolution, the mechanisms of overpassing the developmental constraints, and the origins of complexity and major evolutionary innovations and morphological novelties. I will now examine them in more detail.

## TUMOR-BEARING ORGANISMS AS TRANSITIONAL FORMS IN PROGRESSIVE EVOLUTION

**Fig. 2:**
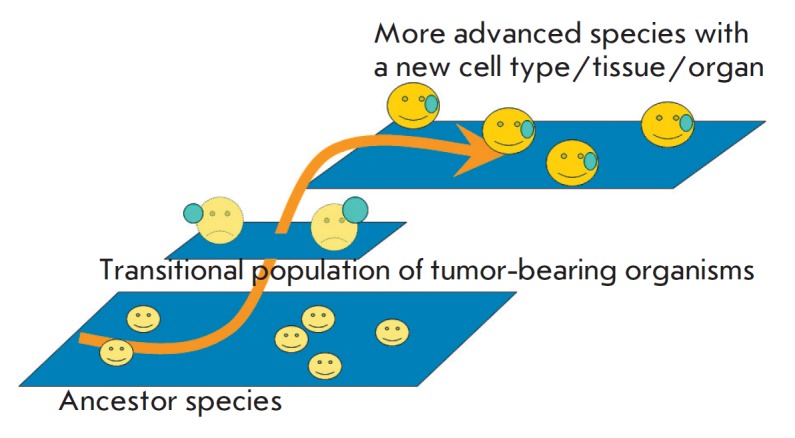
Population of tumor-bearing organisms with heritable tumors as transition between established species of organisms at different levels of complexity. Modified from [1], with permission

According to the main hypothesis, tumor-bearing organisms with hereditary tumors could represent relatively unstable transitional forms that linked phyla with different levels of complexity *(Fig. 2)*. Their stabilization was achieved through the expression of novel genes and gene combinations, and the origin of new functions and functional regulatory feedbacks. As we know from physics, the unstable elementary particles (or some unstable transuranium elements) are difficult to observe. In chemistry, the unstable highly reactive transitionary molecules are difficult to observe as well. Similarly, it is difficult to find tumor-like transitionary structures in paleontological records. A.N. Severtsov has already pointed out that this is because periods of complexity growth were rare and of short-duration [4]. I would add that transitional populations of tumor-bearing organisms could be small, and tumors were soft and not well preserved.

The examples of transitional populations of tumor-bearers are tumor-bearing voles and Xiphophorus fishes with melanomas which were discussed in my book [1]. During certain periods of phylogenesis, differentiation of tumor cells in different organisms of these populations could be frequent enough to result in populations of organisms with a new cell type. The organisms with the new cell type would then be selected for their fitness and competitive abilities. Examples of such selection were discussed in the book [1]. New cell types could participate in the formation of new tissues and organs.

## TUMORS AS THE GENERAL MECHANISM TO OVERCOME DEVELOPMENTAL CONSTRAINTS

Developmental constraints are defined as limitations on phenotypic variability caused by structural and other features of the developmental system [5]. Restraints on variant phenotype production include physical, morphological, genetic and phyletic constraints [6, 7]. Developmental constraints seriously restrict evolutionary changes in animals [8]. The body plan at certain stages is so embedded in the organism’s development that any modification may be lethal [9].

But despite the existence of developmental constraints, morphological novelties have been realized in progressive evolution. The mechanisms through which such transitions happen are not completely understood. The existing hypotheses, e.g. the hypothesis of facilitated variation [10], do not explain how it happened.

My main hypothesis explains that tumors may represent a general way to overcome the developmental constraints in evolutionary perspective, although tumors are connected with present-day pathological conditions. The concept of tumors as engines that search for all possible molecular combinations and innovations by cancellation of major restraints and incompatibilities, formulated in my book [1], helps to understand the possible mechanisms of overpassing the developmental constraints.

Tumors as search engines work in the space of possibilities that have not realized themselves yet. The concept of possibility space is being developed in scientific literature [11-13]. The concepts of morphological, phenotypic and genotype space were also used [5, 7]. The "tumors as a search engine" idea gravitates towards the chaos theory and the complexity theory, which looks for the source of complexity in evolution [14, 15].

The molecular basis of search engine is the global hypomethylation of DNA (discussed in the book), increased global transcription activity [16], and dysregulated transcriptional programs ("transcriptional addiction" [17]) in tumor cells. Gene competition and antagonistic relations between the genes [18, 19] may change significantly in tumor cells due to additional space and resources there. As a result, many unusual genes not expressed in normal cells, including evolutionarily novel genes, are expressed in tumors [2]. Thus, developmental constraints and the compatibility/incompatibility issues are completely or partially abandoned, and unrealized developmental potential is fulfilled.

For morphological innovations, not only novel genes and gene combinations are necessary, but also additional cell masses. According to the main hypothesis, valuable coincidences of unusual gene expression and cell proliferation, which may incidentally happen in tumors, are frozen by natural selection ("frozen accidents" discussed in the book [1]), and lead to the origin of morphological novelties.

Thus, tumors may represent a general mechanism of evolvability of complex organisms and/or developmental plasticity in evolution (see [10, 20] for evolvability and [21, 22] for developmental plasticity). In particular, tumors may facilitate new combinations of "core components" of J. Gerhart and M. Kirschner [10], and/ or core regulatory genes of G. Wagner [23], as well as expression of evolutionarily novel genes. On the contrary, anti-cancer selection may be the source of developmental and evolutionary constraints [24].

## TUMORS AND THE ORIGIN OF NEW CELL TYPES

The number of cell types in Metazoa increased during evolution and may be a measure of their complexity [19, 25]. That is why scientists were looking for the mechanisms of the origin and evolution of new cell types.

The main hypothesis suggests that evolutionarily novel genes and gene combinations are expressed in tumor cells and give rise to a new function and a new regulatory feedback loop. The new function is selected for its enhancement, which also enhances the regulatory feedback. This leads to differentiation of tumor cells in the novel direction and the origin of a new cell type. The new cell type is inherited due to the mechanisms similar to those in preexisting cell type (see discussion in the book [1]). The evolutionary role of cellular oncogenes might consist in sustaining a definite level of autonomous proliferative processes in evolving populations of multicellular organisms and in promoting the expression of evolutionarily new genes. After the origin of a new cell type, the corresponding oncogene should have turned into a cell type-specific regulator of cell division and gene expression [26, 27]. Non-trivial predictions that follow from such a scenario were confirmed in my lab and discussed in the previous paper [3].

The "sister-cell-type model" suggests that novel cell types arise as pairs (sister cell types) from an ancestral cell type by sub-specialization at the last stages of differentiation [28]. This hypothesis works best in the case of terminally differentiated cells but has difficulties in explaining developmental cell types like neural crest-derived cells [23]. In later publication, the authors formulated the "serial sister cell type" hypothesis: "It is now well established that early animal evolution involved the repeated subdivision of the animal body into distinct regions. We propose that these regionalization events also led to the duplication and subsequent diversification of at least one of the cell types that populated that region. This process produced an iterated series of topographically separate sister cell types that we refer to as serial sister cell types. It is plausible that these cell type duplication events also led to the evolution of serial sister stem cells, as virtually all animal cell types co-occurring in one region develop from asymmetrically dividing, multipotent stem cell-like cells" [29]. This hypothesis may also be called "evolution by cell type duplication". It does not contradict my main hypothesis but even converges with it. The pre-existing cell type is under control of natural selection. It is also under regulatory control in the organism. The duplication of a cell type means the origin of extra cells, which escape selection and regulatory control, like in case of gene duplication. The uncontrolled extra cell mass is a neoplasm by definition, which brings the serial sister cell type hypothesis close to the hypothesis of evolution by tumor neofunctionalization.

In tumors, the combination of genes expressed in unrelated or distantly related cell types may be transcribed. In this case, the new cell type will not be in hierarchical relationships predicted by sister-cell-type model. The origin of many cell types from neural crest may be explained in this way. Evolutionarily novel genes expressed in tumors may become targets for core regulatory genes (see Core Regulatory Complex, CRC, [23]) of pre-existing cell types. In such cases, the hierarchical relationship may be conserved.

## TUMORS AND THE ORIGIN OF MAJOR EVOLUTIONARY MORPHOLOGICAL NOVELTIES AND COMPLEX EVOLUTIONARY INNOVATIONS

In my book [1], I presented examples when expression of evolutionarily novel genes in tumors was connected with the origin of new organs (placenta in Mammalia and root nodules in Legumes) and new cell types (macromelanophores in Xiphophorus fishes). The mammary gland and the adaptive immune system are new examples of the possible connection with tumors during the origin of new organs and complex evolutionary innovations.

The mammary gland, an evolutionarily novel organ, may represent a neomorphic hybrid, a mosaic organ whose evolution involved the incorporation of characteristics already encoded in the genome but expressed differently by separate populations of skin glands [30]. The mammary gland coopted signaling pathways and genes for secretory products from earlier integumentary structures [31, 32]. The ancestral tumor could be a mechanism for expression of evolutionarily novel gene combinations in breast tissue, as discussed above. A recent study of evolutionarily novel genes in placental mammals also discovered several novel genes expressed in breast tissue [33].

The adaptive immune system (AIS) originated in jawed fishes and represents a major innovation in evolution of complexity [34]. Two macroevolutionary events – the invasion of the RAG transposon and two whole-genome duplications (WGDs) – are believed to determine the relatively rapid ("big bang") emergence of the AIS in jawed vertebrates [35]. But the origin of clonal expansion and clonal selection of lymphocytes, as well as of different immune cell types and organs, is hard to imagine with only the RAG transposon and WGDs hypotheses. The AIS requires large populations of cells for clonal selection and clonal expansion, and these populations of cells could be provided by an cestral tumors. The computer-like search in ancestral tumors for all possible combinations of molecular and cellular events – the search engine – could be a mechanism of the origin of such complicated evolutionary innovation as AIS, with its combinatorial joining of V, D and J elements.

The number of potential Ig/TCR V region is huge, far exceeding the number of available lymphocytes. The expressed repertoire was studied by variety of methods. The conclusion is that the antibody diversity in nonmammalian vertebrates is low, as opposed to mammals, which make the most of this potential [36, 37].

In frogs, the organization and usage of Ig gene loci is similar to that in mammals, but the diversity of antibodies is much smaller, several orders of magnitude less than in mammals. This is due to major difference in cell number and lymphoid organ architecture. There are few cells in the differentiating immune system of frogs, not enough to realize the potential diversity of the VH locus. Tadpoles have less efficient immune response, i.e. skin graft rejection, and lower Ig and TCR diversity. Simpler organization of the lymphoid frog organs, without lymph nodes or germinal centers, results in poor affinity maturation [36-39].

Thus, cell number limitation represented a serious restriction for the evolution of AIS. Coevolution of lymphoid cell compartment with Ig gene loci might involve tumors. Tumors might provide not only combinatorial possibilities, but also the additional cells necessary for clonal expansion and selection, and for building the structure of lymphoid organs. Indeed, true lymphoid tumors have been discovered in frogs [40, 41].

Without tumors, the origin of such combinatorial innovations as mammary gland and AIS is not possible, because of developmental constraints in established organs and ontogenies.

According to the main hypothesis, the origin of a major evolutionary morphological novelty or complex evolutionary innovation cannot happen by saltatory manner, because it needs the coincidence of too many independent events at different levels of organization. The mechanism for saltatory origin of complex structures does not exist. That is why the unstable transitionary state with search engine capabilities – the tumor – is necessary.

## TUMOR-LIKE PROPERTIES OF EVOLUTIONARILY NEW ORGANS AS AN INDICATION OF THEIR ORIGIN FROM TUMORS

Parallels between the normal and neoplastic development result in solid tumors with many features of normal organs (atypical tumor organs, [42]), on one side, and some normal organs with features of tumors, on the other side.

Normal organs that have features of tumors may be called tumor-like organs. In my book [1], I examined such tumor-like organ, the placenta. Many tumor-like features of placenta were reviewed, and relation of its origin to recurrent germline retrovirus infection was analyzed. The conclusion was drawn that the placenta may be considered a regulated tumor-like organ. After publication of the book, several reviews have been published that basically confirm this point of view [43-45]. Thus, the placenta is a tumor-like organ, first identified in the literature as such.

The developing mammary gland demonstrates many of the properties associated with tumors, e.g. invasion. Terminal end buds (TEBs), a rapidly proliferating mass of epithelial cells, invades into stromal tissue much like a solid tumor [46]. The mammary gland is an evolutionarily young organ. The evolutionary novelty of the mammary gland may be a reason for higher incidence of breast cancer as compared to cancer incidences in evolutionarily older organs [47].

Like the mammary gland, the prostate gland demonstrates correlation of evolutionary novelty with the highest incidence of cancer [47]. Genes differentially expressed in prostate cancer progression overlap with the genes expressed at the earliest stages of prostate development [48]. This indicates the tumor-like nature of the prostate gland.

The common features of tumor-like organs (placenta, mammary gland and prostate) is the presence of the regulated invasion stage in their organogenesis, and the young evolutionary age of these organs. The mammary gland and prostate also demonstrate the highest incidence of cancer. The main hypothesis suggests that atypical tumor organs can give rise to normal organs in evolution, with tumor-like organs as transitional phase.

## TUMORS AND THE GROWTH OF COMPLEXITY

According to the main hypothesis, tumors may be not a consequence, but a prerequisite of the growth of complexity, by providing the building material – extra cells – for expression of evolutionarily novel genes and gene combinations. As it is evident from the above discussion of the origin and evolution of the adaptive immune system (AIS), the access to additional cells necessary for this evolution was not a trivial problem, e.g. for amphibians with their available cell types and stem cells. This problem was resolved in the line Amphibia – Mammalia, with the help of hereditary tumors and tumor stem cells, as suggested by the main hypothesis. With the origin of new functions, atypical tumor-like organs could be stabilized by functional feedbacks, accumulate larger proportion of cells differentiated in new directions and become new organs. The origin of complex organs such as the mammary gland and complex systems such as the AIS may be explained with the help of the "tumors as a search engine" concept discussed above: tumors search for unrealized possibilities in the gene expression possibility space and in the morphological possibility space. Thus, the "tumors as a search engine" concept suggests that chaotic neoplastic development may be a source of complexity in evolution, similarly to suggestion of the dynamical systems theory [14, 15].

## TUMORS AND EMBRYONIC DEVELOPMENT

Normal embryonic development and tumorigenesis have many common features, e.g. invasiveness and cell migration, expression of certain genes and signaling pathways, epithelial-mesenchymal transition, etc.

These commonalities are usually explained by re-activation or deregulation of embryonic signaling pathways in tumors [48-52]. On the other hand, many signaling pathways connected with normal development were first discovered as protooncogenes and tumor-suppressor genes. The terminology of "convergence" of embryonic and tumor signaling pathways is also used (e.g. [53]).

Common features of embryonic development and tumorigenesis are described by several recognized theories. The "embryonal rest" or "embryonic remnants" theory of cancer, formulated over a hundred years ago, suggested that tumors may originate from embryonic cells [54, 55]. This theory was finally proved last year by the results of single-cell transcriptome analysis: the transcriptomes of childhood Wilms tumor cells matched to those of specific fetal cell types [56].

The loss of differentiated functions (e.g. due to mutations) causes tumors. On the other hand, tumor cells can differentiate with the loss of malignancy. This and similar evidence constituted the basis of the differentiation theory of cancer. The more recent stem cell theory of cancer interconnects cancer, cell differentiation, and embryonic development.

The similarities between normal development and tumorigenesis suggest that tumors could participate in the evolution of ontogenesis and in the origin of new cell types, tissues and organs. If true, it explains all the above similarities.

## TUMORS AND EVO-DEVO

A.N. Severtsov defined the following major ways of the evolution of ontogenesis, or modes of phylembryogenesis, as he called them: archallaxis (the change in original anlages), when changes were introduced at the earliest stages of organ embryonic development, or *de novo *formation of evolutionarily new organ occurred; deviation, when the changes were introduced in the intermediary stages of organ embryogenesis; and anaboly, when changes were added at terminal stages of organ ontogenesis, i.e. addition of final stages of morphogenesis [57-59] (see also [60] for review].

From the discussion above it is evident that evolutionarily novel tumor-like organs (placenta, mammary gland, and prostate) represent examples of true archallaxis. The neural crest with its tumor-like cells, recapitulating those of prototype tumor-like formations in early vertebrates [1], may be another example of archallaxis (some researchers consider the neural crest to be a fourth germ layer [61]). Thus, tumors may be a mechanism of the origin of phylogenetically new formations. A.N. Severtsov wrote that unregulated embryonic changes at the earliest stages of organ development produce material for archallaxis, and archallaxis is the most rapid mode of evolution of development [59]. This agrees with the main hypothesis. It is interesting that A.N. Severtsov used the term "new formations," like oncologists did, and claimed that phylogenetic new formations originated by the archallaxis mode.

The origin of the neocortex in humans, related to tumor-like processes as discussed in my book [1], may be connected to deviation and/or anaboly modes. An interesting example of deviation was discussed by A.N. Severtsov in his classical "Morphological Laws of Evolution" [59]: the evolution of nasal pits in Osteichthyes. In *Belone acus*, there is a serious deviation in development of its olfactory pit, which consists in formation of the large mushroom-like outgrowth at the bottom of the pit. The development of this outgrowth resembles tumor growth.

Embryonic, fetal, infantile, and adult tumors, the possible candidates for playing a role in evolution, could participate in evolution of ontogenesis at its different stages. This assumption predicts recapitulations of some tumor features in the most recently evolved organs. Indeed, evolutionarily young organs (placenta, mammary gland, and prostate) recapitulate features of tumors such as invasiveness, the capability of indefinite growth (prostate), the high rates of cancer incidence (mammary gland and prostate), etc.

Thus, tumors may participate in evolution of ontogenesis. Participation of hereditary tumors in evolution of ontogenesis and in the origin of major evolutionary morphological novelties, or phylogenetic new formations, may become an integral part of evolutionary developmental biology, and may be called *carcino-evo-devo*.

## CARCINO-EVO-DEVO, A NEW THEORY OF EVOLUTIONARY DEVELOPMENTAL BIOLOGY

A broad spectrum of non-trivial explanations and non-trivial predictions in different fields of biology, suggested by the main hypothesis, is an indication of its fundamental nature and the potential to become a new biological theory, a theory of the role of hereditary tumors in evolution of development, or *carcino-evo-devo*. Evidently, this abbreviation stems from two other abbreviations – *carcinoembryonic *and *evo-devo *– related to two big areas or research that have brought to formulation of the main hypothesis.

**Fig. 3:**
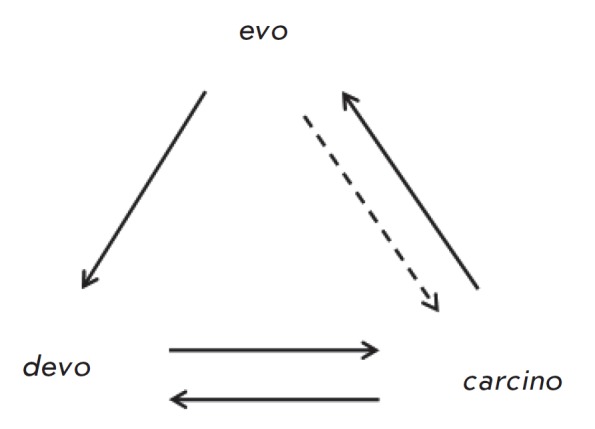
*Carcino-evo-devo *diagram: *devo *– normal ontogenies, *carcino *– ontogenies with neoplastic development, *evo *– progressive evolution of ontogenies. Arrows indicate participation in the corresponding process, or essential connections

The interrelationships between the processes of progressive evolution, normal and neoplastic development may be presented as a diagram *(Fig. 3)*. This diagram represents the relationships between normal ontogenesis and neoplastic development (*devo *↔ *carcino*); participation of hereditary tumors in progressive evolution (*carcino *→ *evo*); and generation of more complex ontogenies in the course of progressive evolution (*evo *→ *devo*). This diagram shows that normal ontogenies do not directly participate in progressive evolution (i.e., the lack of *devo *→ *evo *arrow), and evolution can influence neoplastic development (e.g. anti-cancer selection, dashed arrow between *evo *and *carcino*).

**Fig. 4:**
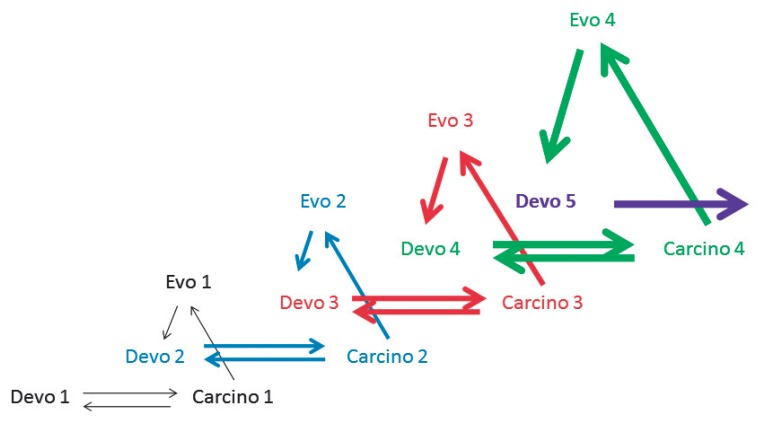
*Carcino-evo-devo *diagrams showing four successive steps in progressive evolution of ontogenesis with tumor participation. Devo 2, Devo 3, Devo 4 and Devo 5 – ontogenies with evolutionarily new progressive traits

According to the *carcino-evo-devo *theory, tumor-bearing organisms participate in progressive evolution that generates new more complex ontogenies. In *Fig. 4*, four *carcino-evo-devo *diagrams show successive steps in progressive evolution of ontogenesis leading to the origin of different morphological novelties and complex evolutionary innovations, with participation of tumors.

The *carcino-evo-devo *diagram reminds the central dogma of molecular biology not only in its outward appearance. Like the central dogma, it contains a fundamental prohibition: a prohibition of saltatory origin of complex evolutionary innovations and morphological novelties directly from normal ontogenies. As I wrote above, the mechanisms of saltatory origin of complex structures do not exist. The *carcino-evo-devo *theory demands the necessity of transitionary intermediates with search engine capabilities, which I think are tumor-bearing organisms (*carcino*). I hope that the *carcino-evo-devo *diagram will cause discussion on what the transitionary intermediates should be, and on the number of arrows and their possible directions.

Thus, a new theory of the possible role of hereditary tumors in evolution – *carcino-evo-devo *– is being developed. This theory possesses a predictive power, explains many previously unexplained biological phenomena, accommodates a large amount of data, and has a potential of unifying several existing biological theories. It may become a new theory of evolutionary developmental biology.

## Abbreviations

AIS: adaptive immune system
CRC: Core Regulatory Complex
C/T antigens: cancer/testis antigens
RAG: recombination-activating genes
TCR genes: T-cell receptor genes
TEBs: Terminal end buds
WGDs: whole-genome duplications

## References

[1] Kozlov A.P. (2014). Evolution by Tumor Neofunctionalization. The role of tumors in the origin of new cell types, tissues and organs. Amsterdam, Boston, Heidelberg, London, New York, Oxford, Paris, San Diego, San Francisco, Singapore, Sydney, Tokyo: Elsevier/Academic Press, 2014. 231 p..

[2] Kozlov A.P. (2016). Infect. Agents Cancer..

[3] Makashov A.A., Malov S.V., Kozlov A.P. (2019). Sci. Rep..

[4] Severtsov A.N. (1925). The main directions of the evolutionary process. Moscow: Dumnov Publishing House, 1925..

[5] Maynard Smith J., Burian R., Kauffman S., Alberch P., Campbell J., Goodwin B., Lande R., Raup D., Wolpert L. (1985). The Quarterly Review of Biology..

[6] Gilbert S.F. (2000). Developmental Biology, 6th edition. Sunderland (MA): Sinauer Associates Inc.,U.S. 2000. 695 p..

[7] Wagner A. (2011). Origins of Evolutionary Innovations. Oxford, UK: Oxford University Press, 2011. 253 p..

[8] Galis F., Metz J.A.J., von Alphen J.J.M. (2018). Annual Reviews of Ecology, Evolution and Systematics..

[9] Raff R.A. (1996). The Shape of Life. Chicago and London: Univ. of Chicago Press, 1996. 544 p..

[10] Gerhart J., Kirschner M. (2007). Proc. Natl. Acad. Sci. USA..

[11] Popper K. (1991). Advances in Scientific Philosophy. Amsterdam: Rodopi, 1991..

[12] Katsman R. (2013). Literature, history, choice: The Principle of Alternative History in Literature (S.Y. Agnon, The City with All That is Therein). Newcastle upon Tyne, UK: Cambridge Scholars Publishing, 2013. 30 p..

[13] Rescher N. A. (2015). Journey through philosophy in 101 anecdotes. Pittsburgh, PA: University of Pittsburgh Press, 2015. 304 p..

[14] Kaneko K. (1994). Artificial Life..

[15] Warren K. (2013). Chaos theory and complexity theory. Encyclopedia of Social Work. Oxford, UK: Oxford University Press, 2013..

[16] Kotsantis P., Marques Silva L., Irmscher S., Jones R.M., Foles L., Gromak N., Paterman E. (2016). Nature Communications..

[17] Bradner J.E., Hnisz D., Young R.A. (2017). Cell..

[18] Kozlov A.P. (1976). Regulatory mechanisms as an expression and the result of evolution of competitive relations between the genes. In: Explorations of the Fauna of the Seas 17 (25). Salinity Adaptations of the Aquatic Animals. Leningrad: Academy of Sciences of the U.S.S.R., 1976..

[19] Kozlov A.P. (1979). J. Theor. Biol..

[20] Kirschner M., Gerhart J. (1998). Proc. Natl. Acad. Sci. USA..

[21] West-Eberhard M.J. (2003). Developmental plasticity and evolution. New York: Oxford University Press, 2003. 814 p..

[22] Moczek A.P. (2015). Heredity..

[23] Wagner G.P. (2014). Homology, genes, and evolutionary innovation. Princeton: Princeton University Press, 2014. 496 p..

[24] Galis F., Metz J.A.J. (2003). BioEssays..

[25] Valentine J.W., Collins A.G., Meyer C.P. (1994). Paleobiology..

[26] Kozlov A.P. (1987). Theoretical and Mathematical Aspects of Morphogenesis. Moscow: Nauka, 1987..

[27] Kozlov A.P. (1996). Med. Hypotheses..

[28] Arendt D. (2008). Nat. Rev. Genet..

[29] Arendt D., Musser J.M., Baker C.V.H., Bergman A., Cepko C., Erwin D.H., Pavlicev M., Schlosser G., Widder S., Laubichler M.D., Wagner G.P. (2016). Nature Rev. Genet..

[30] Blackburn D.G. (1991). Mammal. Rev..

[31] Vorbach C., Capecchi M.R., Penninger J.M. (2006). BioEssays..

[32] Oftedal O.T. (2002). J. Mammary Gland Biol. Neoplasia..

[33] Dunwell T.L., Paps J., Holland P.W.H. (2017). Proc. R. Soc. B.

[34] Muller V., de Boer R.J., Bonhoeffer S., Szathmany E. (2018). Biol. Rev..

[35] Flajnik M.F., Kasahara M. (2010). Nat. Rev. Genet..

[36] Du Pasquier L. (1993). Fundamental immunology, 3rd edition. New York: Raven Press, 1993..

[37] Flajnik M., Miller K., Du Pasquier L. (2003). Fundamental immunology. Philadelphia: Lippincott, Williams and Wilkins, 2003..

[38] Du Pasquier L., Robert J., Courtet M., Mussmann R. (2000). Immunol. Rev..

[39] Robert J., Ohta Y. (2009). Dev. Dyn..

[40] Robert J., Cohen N. (1998). Immunol Rev..

[41] Goyos A., Robert J. (2009). Front. Biosci..

[42] Egeblad M., Nakasone E.S., Werb Z. (2010). Dev. Cell..

[43] Kurlak L.O., Knofler M., Mistry H.D. (2017). Placenta..

[44] Costanzo V., Bardelli A., Siena S., Abrignani S. (2018). Open Biol..

[45] Bronchud M.H. (2018). Ecancermedicalscience..

[46] Wiseman B.S., Werb Z. (2002). Science..

[47] Davies J.A. (2004). Organogenesis..

[48] Schaefer E.M., Marchionni L., Huang Z., Simons B., Blackman A., Yu W., Parmigiani G., Berman D.M. (2008). Oncogene..

[49] Ma Y., Zhang P., Wang F., Yang J., Yang Z., Qin H. (2010). J. Cell Mol. Med..

[50] Micalizzi D.S., Farabaugh S.M., Ford H.L. (2010). J. Mam. Gland Biol. Neopl..

[51] Aiello N.M., Stanger B.Z. (2016). Dis. Model Mech..

[52] Kohrman A.Q., Matus D.Q. (2017). Trends Cell Biol..

[53] Hniz D., Schuijers J., Lin C.Y., Weintraub A.S., Abraham B.J., Lee T.I., Bradner J.E., Young R.A. (2015). Molecular Cell..

[54] Durante F. (1874). Arch. Memor. Observ. Chir. Prat..

[55] Cohnheim J. (1889). Lectures on General Pathology, vol. 2. London: The New Syndenham Society, 1889. 1434 p..

[56] Young M.D., Mitchell T.J., Vieira Braga F.A., Tran M.G.B., Stewart B.J., Ferdinand J.R., Collord G., Botting R.A., Popescu D.M., Loudon K.W. (2018). Science..

[57] Severtsov A.N. (1927). Jena Z. Naturwiss..

[58] Severtsov A.N. (1935). Zool. Zhur..

[59] Severtsov A.N. (1949). Morphological Laws of Evolution. Collection of works, vol. 5. Moscow, Leningrad: Publishing House of the Academy of Sciences of the U.S.S.R., 1949..

[60] Gould S.J. Ontogeny and Phylogeny. Cambridge: Belknap Press: An Imprint of Harvard University Press, 1977. 520 p..

[61] Hall B.K. (2000). Evolution & Development..

